# Dihydroartemisinin-Loaded Chitosan Nanoparticles Inhibit the Rifampicin-Resistant *Mycobacterium tuberculosis* by Disrupting the Cell Wall

**DOI:** 10.3389/fmicb.2021.735166

**Published:** 2021-09-22

**Authors:** Xiujuan Gu, Qi Cheng, Ping He, Yan Zhang, Zhengfang Jiang, Yali Zeng

**Affiliations:** ^1^Department of Clinical Laboratory, Affiliated Hospital of Southwest Medical University, Luzhou, China; ^2^Department of Clinical Laboratory, Sichuan Mianyang 404 Hospital, Mianyang, China; ^3^Respiratory Medicine, Chengdu Seventh People’s Hospital, Chengdu, China; ^4^School of Materials Science and Engineering, Southwest University of Science and Technology, Mianyang, China

**Keywords:** *Mycobacterium tuberculosis*, rifampin-resistance, dihydroartemisinin, chitosan, nanoparticle, gas chromatography-mass spectrometry, metabolomics

## Abstract

Tuberculosis (TB) caused by *Mycobacterium tuberculosis* (MTB) is a deadly infection, and increasing resistance worsens an already bad scenario. In this work, a new nanomedicine antibacterial agent, based on dihydroartemisinin (DHA) and chitosan (CS), has been successfully developed to overcome MTB’s drug-resistant. To enhance DHA’s solubility, we have prepared nanoparticles of DHA loaded CS by an ionic crosslinking method with sodium tripolyphosphate (STPP) as the crosslinking agent. The DHA-CS nanoparticles (DHA-CS NPs) have been fully characterized by scanning electron microscopy, Fourier transforms infrared spectroscopy, dynamic light scattering, and ultraviolet spectrophotometry. DHA-CS NPs show an excellent antibacterial effect on the rifampicin (RFP)-resistant strain (ATCC 35838) and, at a concentration of 8.0 μg/ml, the antibacterial impact reaches up to 61.0 ± 2.13% (*n* = 3). The results of Gram staining, acid-fast staining, auramine “O” staining and electron microscopy show that the cell wall of RFP-resistant strains is destroyed by DHA-CS NPs (*n* = 3), and it is further verified by gas chromatography-mass spectrometry. Since all the metabolites identified in DHA-CS NPs treated RFP-resistant strains indicate an increase in fatty acid synthesis and cell wall repair, it can be concluded that DHA-CS NPs act by disrupting the cell wall. In addition, the resistance of 12 strains is effectively reduced by 8.0 μg/ml DHA-CS NPs combined with RFP, with an effective rate of 66.0%. The obtained results indicate that DHA-CS NPs combined with RFP may have potential use for TB treatment.

## Introduction

Caused by *Mycobacterium tuberculosis* (MTB), tuberculosis (TB) is a chronic infectious disease with high mortality rate. TB incidence has been gradually increasing in recent years, and it has become a significant global public health problem (Stein et al., 2008). According to the worldwide progress report on tuberculosis elimination 2020, TB still accounts for the highest mortality from any infectious diseases worldwide, causing 1.5 million deaths in 2018. In addition, approximately half a million new cases of rifampicin (RFP)-resistant TB were reported with 78.0% multidrug-resistant TB (MDR-TB) in 2018 (Harding, 2020). According to the global tuberculosis report 2019, the latest treatment data has shown that the treatment success rate of MDR-TB is 56.0%, and extensively severe drug-resistant TB (XDR-TB) is almost impossible to incurable. Both MDR-TB and XDR-TB are resistant to RFP. Therefore, developing anti-TB new drugs is a practical approach to the treatment of RFP-resistant TB (Dheda et al., 2014; Maitre et al., 2017).

Artemisinin is one of sesquiterpene lactone peroxides isolated from the Chinese plant *Artemisia annua* L. Multiple derivatives with active metabolite dihydroartemisinin (DHA) have been developed with improved pharmacological features (Zhu, 2014). The pharmacological effects of DHA mainly include antimalarial, antitumoral, antischistosomal, and antibacterial activities (Meshnick et al., 1993; Want et al., 2015). Recent studies have confirmed that artemisinin derivatives have bacteriostatic effects on MTB (Zhang et al., 2014; Beg et al., 2017; Upadhyay et al., 2017; Wais et al., 2017). However, artemisinin derivatives have also disadvantages such as poor water solubility, low bioavailability, short duration of effective blood drug concentration and rapid excretion (Jin, 2020). Nowadays, no in-depth studies have been conducted on the application of artemisinin drugs for TB prevention.

Nanomedicine refers to the use of nanotechnology to prepare drugs and corresponding carriers into a new class of pharmaceuticals with a particle size ranging from 1 to 1,000 nm (Satalkar et al., 2016). It can improve drug absorption and drug efficacy, increase drug stability, enhance drug targeting, and reduce drug toxicity (Rani et al., 2018; Kumari et al., 2019; Martinelli et al., 2019; Wang et al., 2019). CS is a natural polymer with peculiar benefits, such as biocompatibility, biodegradability, non-toxicity, mucoadhesion and antibacterial properties, and can activate macrophages to increase the bactericidal activity (Hou and Zhao, 2006; Kucukoglu et al., 2019; Radwan-Pragłowska et al., 2019). MTB can persist in an immune-competent host via several immune evasion strategies, such as altered antigen presentation to prevent the recognition of infected macrophages by T cells, thereby evading macrophage killing mechanisms (Sia and Rengarajan, 2019). Therefore, chitosan is expected to become an effective carrier for the treatment of TB.

In this work, DHA-CS nanoparticles (DHA-CS NPs) were prepared using CS as the drug carrier to investigate the antibacterial activity of DHA-CS NPs on RFP-resistant strains and the mechanism of action. It would lay a substantial foundation for the clinical application of DHA-CS NPs.

## Materials and Methods

### Bacterial Culture

Eighteen clinical drug-resistant strains and one RFP-resistant MTB strain (ATCC 35838) were obtained from Mianyang Centers for Disease Control and Prevention (CDC), China, and stored in a refrigerator at –80°C. The information about strains is shown in Table 1. MTB strains were inoculated on L-J medium (Shanghai Yiheng Scientific Instruments Co., Ltd., Shanghai, China) and cultured at 37°C in a 5% CO_2_ incubator (Zhou et al., 2017).

**TABLE 1:** Information on strains.

Serial number	Name	Source	Drug-resistance
1	Clinical isolates	Mianyang CDC	RFP
2	Clinical isolates	Mianyang CDC	RFP
3	Clinical isolates	Mianyang CDC	RFP
4	Clinical isolates	Mianyang CDC	RFP, INH, EMB
5	Clinical isolates	Mianyang CDC	RFP, INH
6	Clinical isolates	Mianyang CDC	RFP
7	Clinical isolates	Mianyang CDC	RFP, INH, EMB
8	Clinical isolates	Mianyang CDC	RFP
9	Clinical isolates	Mianyang CDC	RFP
10	Clinical isolates	Mianyang CDC	RFP
11	Clinical isolates	Mianyang CDC	RFP, INH
12	Clinical isolates	Mianyang CDC	RFP
13	Clinical isolates	Mianyang CDC	RFP
14	Clinical isolates	Mianyang CDC	RFP
15	Clinical isolates	Mianyang CDC	RFP
16	Clinical isolates	Mianyang CDC	RFP, INH
17	Clinical isolates	Mianyang CDC	RFP
18	Clinical isolates	Mianyang CDC	RFP, INH
19	ATCC35838	Mianyang CDC	RFP

### Materials

DHA was purchased from Ron Reagent Company (Shanghai, China). CS with molecular weight 50–190 kDa (degree of deacetylation > 95.0%) was purchased from Guanghan Hengyu New Materials Co., Ltd. (Chengdu, China). Sodium tripolyphosphate (STPP) was provided by Southwest University of Science and Technology, Sichuan, China.

### Preparation of DHA-CS NPs

DHA-CS NPs were produced using ionic-gelation methods with STPP. 50.0 mg CS was dissolved in 50 ml 1.0% acetic acid solution. 10.0 mg DHA and 10.0 mg STPP were dissolved in 10 ml absolute ethanol. Then, DHA-STPP ethanol solution was slowly dripped into the CS solution with magnetic stirring at 1,000 rpm and stirred at 60°C for 2 h. CS NPs were prepared with the same method.

### Morphology and Particle Size and FTIR Spectrum

DHA-CS NPs were diluted to 0.50 μg/ml using distilled water. The morphology of DHA-CS NPs was observed by scanning electron microscope (SEM) (Ultra 55, ZEISS, Heidenheim, Germany). Particle sizes and dispersion index of DHA-CS NPs were analyzed using a laser particle size analyzer (90 plus, Brookhaven, Waltham, MS, United States). Fourier transform infrared (FTIR) spectrometer (Nicolet 6700, Thermo Fisher Scientific, Waltham, MA, United States) was used to identify the chemical compounds of samples.

### Entrapment Efficiency and Drug Loading

10.0 mg DHA was added into a 100 ml volumetric flask and made up to 100 ml with 2.0% NaOH aqueous solution and ethanol (4:1, v/v). The DHA solution was incubated for 2 h to obtain a 100.0 μg/ml standard solution. 0.20, 0.60, 1.0, 1.4, 1.8 ml standard solutions were put into different volumetric flasks (10 ml) and then heated in a water bath at 60°C for 30 min. The absorbance of sample solutions was measured in the wavelength range of 200–400 nm by an ultraviolet spectrophotometer (Evolution 300, Thermo Fisher Scientific, Waltham, MA, United States). Then, the linear regression of DHA concentration (C) with DHA absorbance (A) was obtained.

500 μl DHA-CS NPs were placed into an ultrafiltration centrifuge tube (Beijing Cedoris Scientific Instruments Co., Ltd., Beijing, China) and centrifuged at 3,340 *g* (TGL-18M, BIOBASE, Lu Xiangyi Centrifuge Co., Ltd., Shanghai, China) for 15 min. Equal amounts of ultrafiltration solutions and non-centrifuged DHA-CS NPs were placed into different volumetric flasks (10 ml) and made up to 10 ml with 2.0% NaOH aqueous solution and ethanol (4:1, v/v). The two volumetric flasks were incubated in a water bath at 60°C for 30 min. Finally, the absorbance of free DHA in centrifuged and non-centrifuged DHA-CS NPs solutions was measured. The entrapment efficiency and drug loading of DHA-CS NPs were calculated as follows:

Entrapment efficiency (%) = (total mass of DHA—mass of free DHA)/total mass of DHA × 100% (1)

Drug loading (%) = (total mass of DHA—mass of free DHA)/total mass of nano-DHA composite × 100% (2)

### Preparation of Bacterial Suspension

3–4 drops of 0.50% Tween 80 and resistant MTB colonies grown on L-J medium for 3–4 weeks were placed into a thick large glass tube. After grinding, 6–8 drops of physiological saline were dropped into the tube and then stood to precipitate large particles of bacteria. Finally, supernatants were aspirated, and Middlebrook 7H9 Broth Base (Sigma, San Francisco, California, United States) was used to adjust the supernatants to 3.0 × 10^8^ CFU/ml.

### Optimal Concentration of DHA-CS NPs

The antimicrobial effect of 0.50–256.0 μg/ml drugs on ATCC 35838 MTB was detected by bacterial dead/live ratio testing, and an appropriate drug concentration was selected for the other experiment. Different concentrations (0.50, 1.0, 2.0, 4.0, 8.0, 16, 32, 64, 128, 256 μg/ml) of drugs (DHA-CS NPs, CS NPs, and DHA) and bacterial solutions [diluted to 3.0 × 10^7^ CFU/ml, referenced MTB liquid drug sensitivity kit instructions (Autobio, Shanghai, China)] were added at 100.0 μl/well in 96-well plates (Kangborui Biological Technology Co., Ltd., Chengdu, China). Three parallel holes were set up for each concentration. Negative and blank control groups were established simultaneously and incubated in an incubator at 37°C for 6 days. Then, samples were stained according to the instructions of LIVE/DEADTM BacLight^TM^ Bacterial Viability Kit reagent (Invitrogen, San Francisco, California, United States). The FSC LOG and SSC LOG parameters were selected to establish FSC LOG VS. SSC LOG scatter plots, and the gain and voltage were adjusted to make sure all events are in the plot. Sterile 7H9 solution was used as a blank control to deduct the background noise. Then we used individually stained live bacteria and dead bacteria to adjust the FL1, FL3 voltages, and appropriate compensation value. Finally, the flow cytometry (FC500, Beckman, Waltham, MA, United States) scheme was formulated, and live and dead bacteria areas were delimited (Wu, 2015).

Antibacterial rates were calculated as follows: Antibacterial rate (%) = (number of live bacteria in the control group − number of live bacteria in the experimental group)/number of live bacteria in the control group × 100%.

Then, the optimal concentration for the next experiments was selected by bacterial live/dead ratio testing.

Group-wise differences were tested by one-way ANOVA or *t*-test using SPSS 17.0, and *p*-value < 0.05 was considered to be statistically significant.

### Auramine “O” Staining, Acid-Fast Staining and Gram Staining

Auramine “O” staining, acid-fast staining and Gram staining (Baso, Zhuhai, China) methods were used to observe the number and morphology of ATCC 35838 MTB in the presence and absence of drugs. DHA-CS NPs, CS NPs, and free DHA with the final concentration of 8.0 μg/ml were separately added to bacterial suspensions (3.0 × 10^8^ CFU/ml), and three parallel trials were set up for each drug. The negative control group was established simultaneously. Then, samples were incubated at 37°C in a 5% CO_2_ incubator for 6 days. After that, the suspension was centrifuged for 10 min at 4,000 *g* to remove excess supernatant and retain only 20 μl. After mixing, precipitates were placed on different slides and smeared as a 2 cm diameter circular film. After UV irradiation for 2 h, MTB was subjected to staining. Finally, MTB of auramine “O” staining was observed using a fluorescence microscope at 400× magnification (E 100, Nikon, Tokyo, Japan). MTB of acid-fast staining and Gram staining was observed at 1,000× magnification by oil immersion technique (BX 43, Olympus, Tokyo, Japan).

### SEM

SEM was used to observe ATCC 35838 MTB’s structure in the presence and absence of drugs. DHA-CS NPs, CS NPs and free DHA with the final concentration of 8.0 μg/ml were added to bacterial suspensions (3.0 × 10^8^ CFU/ml), and three parallel trials were set up. The negative control group was established simultaneously. Then samples were cultured at 37°C in a 5% CO_2_ incubator for 6 days.

After centrifuged for 13 min at 300 *g*, supernatants were discarded. 50 μl residues were placed on glass slides and fixed with 3% glutaraldehyde for 30 min, then washed three times with dH_2_O. The slides were air-dried and dehydrated by a graded series of ethanol (30, 50, 70, 80, 90, and 95%) for 15 min at each step, and finally dehydrated two times by absolute ethanol for 20 min. After that, samples were dried ambiently, and the morphology of MTB was observed using field emission SEM.

### Metabolomics Analysis

The influence of DHA-CS NPs on MTB metabolic pathway was detected by metabolic omics method. ATCC 35838 RFP-resistant strain was suspended to approximately 1.0 × 10^7^ colony-forming units CFU/ml (van Breda et al., 2015; Koen et al., 2018). Cell suspensions (2.0 ml) and DHA-CS NPs (final concentrations of 0.0 and 8.0 μg/ml) were added to each 5.0 ml EP tubes. They were incubated at 37°C for 24 h. The three samples containing no DHA-CS NPs and four samples containing 8.0 μg/ml DHA-CS NPs were centrifuged at 10,000 *g* for 1 min, and sediments were collected. 10.0 mg of each sample was placed into an EP tube and extracted with chloroform, methanol and water in a ratio of 1:3:1. Samples were repeatedly placed in liquid nitrogen freeze-thaw 5 times (2 min each time) and sonicated for 30 min in an ice bath, then centrifuged at 10,000 g for 1 min at 4°C. The supernatants were transferred to different sample bottles, and 2.0 μl of 3-phenyl butyric acid (Kangbo Rui Biotechnology Co., Ltd., Chengdu, China) was added as an internal standard (1.75 μg/ml). After freeze-drying, the samples were redissolved in 80 μl methoxyamine hydrochloride in pyridine solution (20.0 mg/ml), and the oximation reaction was performed in a water bath at 50°C for 90 min. Then, methoxyamine hydrochloride-(trimethylsilyl)-trifluoroacetamide (Shanghai Xinyu Biological Technology Co., Ltd., Shanghai, China) derivatization reagent containing 1.0% trimethylchlorosilane (Shanghai Xinyu Biological Technology Co., Ltd., Shanghai, China) was added to each sample. After that, the derivatization reaction was carried out in a 50°C water bath for 90 min (Wang et al., 2015).

Samples (1.0 μl) were analyzed in a random sequence by gas chromatography-mass spectrometer (GC-MS) (TQ8050, Shimadzu Corporation, Tokyo, Japan). Shimadzu HP-5 capillary column (30 m × 250 μm × 0.25 μm) was used for GC separation of compounds. High-purity helium was used as the carrier gas with a carrier gas flow rate of 1.0 ml/min. Interface temperature was 280°C, and inlet temperature was 270°C; initial temperature of column was set to 70°C for 4 min, then raised to 133°C at the rate of 3°C/min, heated to 200°C at the rate of 2°C/min, increased to 220°C and finally heated to 260°C at the rate of 5°C/min. The ionization mode was electron bombardment with an electron energy of 70 eV and a temperature of 230°C. A mass range of 85∼500 m/z was used for the mass spectra.

Mass spectrometry deconvolution, peak alignment and peak identification were carried out on the raw data by GC-MS Solution software (version 2.1). The qualitative analysis was performed by matching with the National Institute of Standards and Technology (NIST). The data were analyzed with SIMCA-P (version 13.0), including principal component analysis (PCA), partial least squares discriminant analysis (PLS-DA) and orthogonal partial least squares discriminant analysis (OPLS-DA). SPSS 17.0 was used to perform *t*-test on the data, and *p*-value < 0.05 was considered statistically significant.

### DHA-CS NPs Combined With RFP

Solid drug sensitivity testing was used to detect whether the combination of DHA-CS NPs and RFP could reduce the resistance of clinical RFP-resistant strains. Bacterial suspensions of eighteen clinical resistant strains (serial number: 1–18) were diluted to 3.0 × 10^6^ CFU/ml and 3.0 × 10^4^ CFU/ml with 0.90% NaCl solution. DHA-CS NPs with final concentrations of 8.0 μg/ml was added to diluted suspensions, and then they were placed at 37°C in a 5% CO_2_ incubator for 24 h. Negative control groups were established at the same time (without DHA-CS NPs). Afterward, bacterial suspensions were inoculated on the surface of drug-sensitive solid culture media (Zhuhai Bezo Biotechnology Co., Ltd, Sichuan, China) at 20.0 μl/well, and placed at 37°C in a 5% CO_2_ incubator for 1 month.

The tangible medium is a plate with multiple culture wells (Supplementary Figure 1). Each culture plate has two wells (“a” and “b”) without drugs, and the remaining wells contain anti-tuberculosis drugs. The wells of “c” and “d” are embedded with isoniazid (INH), “e” and “f” wells are embedded with ethambutol (EMB), “g” and “h” wells are embedded with RFP (in this work, only RFP was concerned). Bacterial suspensions of negative control groups were inoculated in “a” and “b” wells, and the DHA-CS NPs drug groups were inoculated in other wells.

## Results

### Morphology, Particle Size Distribution and Dispersion Index of DHA-CS NPs

**FIGURE 1:**
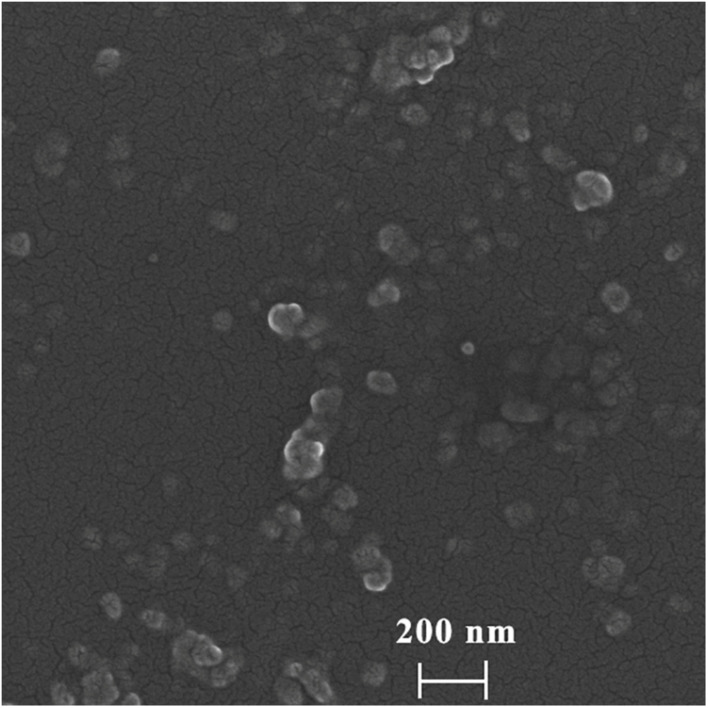
SEM of DHA-CS NPs.

To determine the morphology and particle size of DHA-CS NPs, SEM, and laser particle size analyzer were used. The average particle size of DHA-CS NPs was 217.0 ± 8.12 nm, and the dispersion index was 0.342 ± 0.01 (*n* = 3). SEM results showed that the DHA-CS NPs formed a uniform quasi-spherical shape with low polydispersity, and the diameter was consistent with the above particle size measurement results (Figure 1).

### FTIR Assayed DHA Loading on CS NPs

**FIGURE 2:**
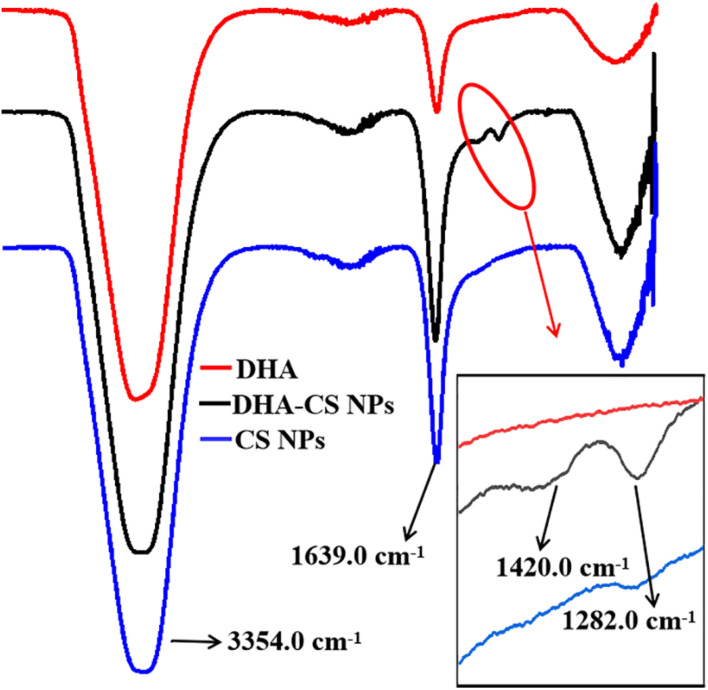
FTIR spectra of DHA-CS NPs, CS NPs, and DHA.

FTIR analysis was used to confirm whether DHA was loaded on CS. DHA, CS NPs, and DHA-CS NPs were aqueous solutions, so the results of FTIR were significantly affected by the characteristic peaks of water. In Figure 2, the bands near 3354.0 cm^–1^ and 1639.0 cm^–1^ are ascribed to the characteristic hydroxyl peaks of adsorbed water (Dinache et al., 2020). The absorption peak intensity of CS NPs and DHA-CS NPs near 1639.0 cm^–1^ was significantly stronger than that of DHA and presented the stretching vibration of C = O in amide bond, indicating the successful crosslinking interaction between TPP and the amino groups of CS within CS NPs (Ye et al., 2015). In addition, compared with CS NPs, two new peaks of DHA-CS NPs near 1326.0 cm^–1^ and 1282.0 cm^–1^ were due to the crosslinking interaction between DHA and TPP, indicating that DHA was successfully loaded on CS NPs.

### Encapsulation Efficiency and Drug Loading Capacity of DHA-CS NPs

**FIGURE 3:**
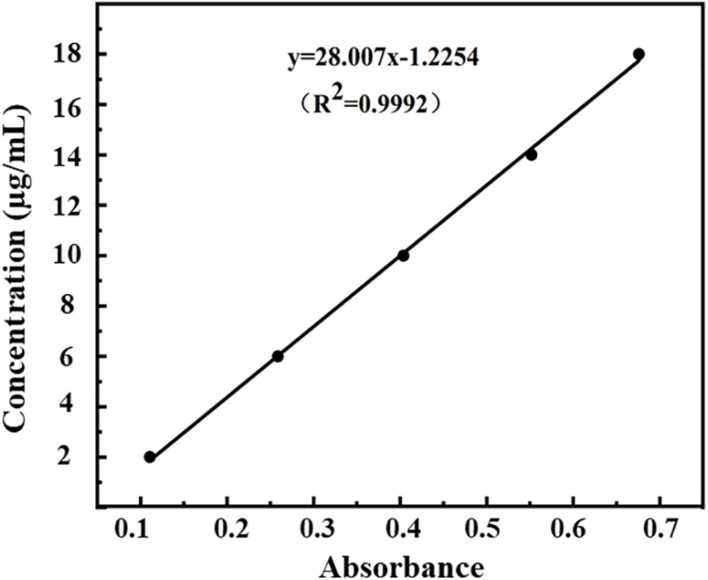
Standard curve of absorbance-concentration of DHA measured at 238 nm by UV-VIS spectroscopy.

The measured optical densities (ODs) of DHA standard solutions were 0.111, 0.259, 0.404, 0.552, and 0.676, and the OD curve was fitted by the regression equation C = 28.01A–1.22 (*R*^2^ = 0.9992, *n* = 5). A good linear correlation was found between DHA concentration (C) and OD (A) in the range of 2.0–18.0 μg/ml (Figure 3).

According to Equations (1) and (2), the encapsulation efficiency of DHA-CS NPs was calculated to be (76.4 ± 4.53)%, and the drug loading capacity was (7.9 ± 0.12)% (*n* = 3). The results above indicated the successful preparation of DHA-CS NPs.

### DHA-CS NPs Had Antibacterial Activity Against MTB

**FIGURE 4:**
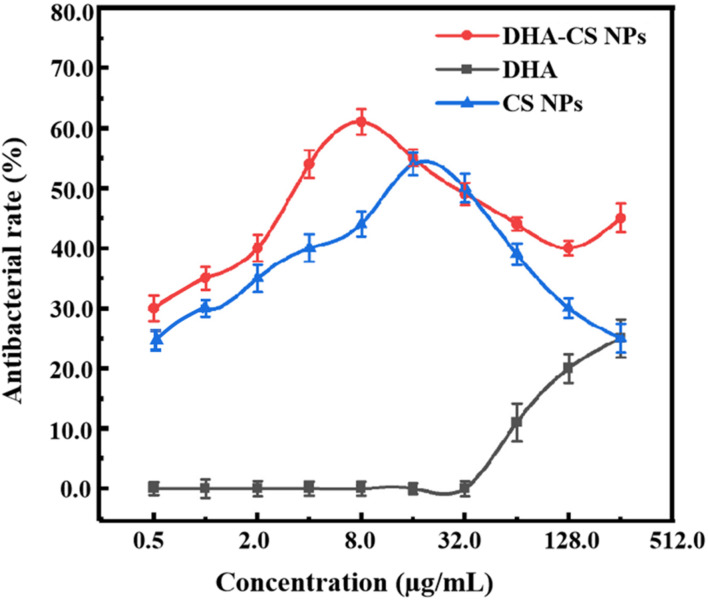
Antibacterial rate curves of MTB treated with 0.50–256 μg/ml drugs for 6 days (*n* = 3).

To detect the antibacterial effect and select DHA-CS NPs concentration, the antibacterial rate curves were drawn using the bacterial dead/live ratio testing. The antibacterial rate curve in Figure 4 showed that enhanced antibacterial activity of DHA was observed with increasing the concentration at the range of 32.0–256.0 μg/ml, and the highest value was obtained at 256.0 μg/ml. However, the DHA concentration of 256.0 μg/ml had no clinical application value (Li et al., 2020).

CS is a broad-spectrum antibacterial agent and has certain antibacterial effects against *Escherichia coli*, *Pseudomonas aeruginosa*, *Enterococcus faecalis*, and *Staphylococcus saprophyticus* (Andres et al., 2007). In this work, CS also showed anti-Mycobacterial results. The highest antibacterial effect of CS NPs was obtained at 16.0 μg/ml with a value of (54.4 ± 1.93)%. Compared with CS NPs, the antibacterial effect of DHA-CS NPs enhanced (*p* < 0.05), and it could be due to an additive antibacterial effect of DHA and CS (Jøraholmen et al., 2020). The antibacterial effect of DHA-CS NPs (0.5–256.0 μg/ml) was highest at 8.0 μg/ml with a value of (61.0 ± 2.13)%. Therefore, the optimal concentration of DHA-CS NPs was selected as 8.0 μg/ml for the next experiments.

### Morphology and Quantity of MTB Changed After DHA-CS NPs Treatment

**FIGURE 5:**
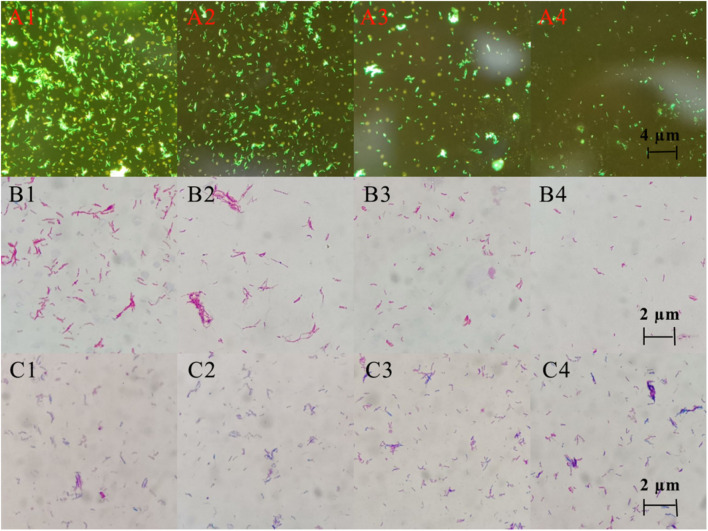
Auramine “O” staining, acid-fast staining, and gram staining pictures of MTB after 6 days treatment with 8.0 μg/ml DHA-CS NPs (*n* = 3). **(A1–4)** auramine “O” staining, **(B1–4)** acid-fast staining, **(C1–4)** Gram staining, **(A1,B1,C1)** Negative control group, **(A2,B2,C2)** DHA drug group, **(A3,B3,C3)** 8.0 μg/ml CS NPs drug group, **(A4,B4,C4)** 8.0 μg/ml DHA-CS NPs drug group.

Three staining experiments were used to detect the morphology and quantity of MTB after drug treatment. In the negative control group and DHA group, acid-fast staining and auramine “O” staining of MTB in Figure 5 showed that bacteria were slightly curved, long rod-shaped, chain-shaped, branched aggregated, and grown. The results of the DHA-CS NPs group and CS NPs group showed that MTB was short rod-shaped and arranged individually; the number of MTB was significantly reduced.

The Gram staining results of MTB showed that it was not easy to stain, and the outline of bacteria was not clear. Compared with the negative control group and the DHA group, Gram staining results showed that the outline of MTB treated with DHA-CS NPs and CS NPs was clear and easily stained.

### Bacterial Wall and Membrane Integrity Was Destroyed by DHA-CS NPs

**FIGURE 6:**
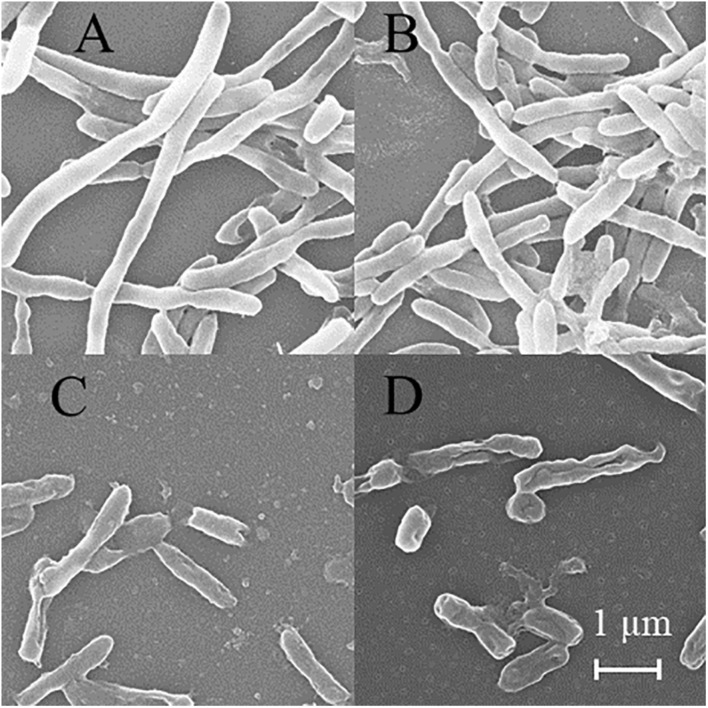
SEM of MTB treated by 8.0 μg/ml DHA-CS NPs for 6 d (*n* = 3). **(A)** negative control group, **(B)** 8.0 μg/ml free DHA drug group, **(C)** 8.0 μg/ml CS NPs drug group, **(D)** 8.0 μg/ml DHA-CS NPs drug group.

SEM was used to detect the structure of MTB after DHA-CS NPs treatment. As shown in Figure 6, the MTB of the negative control group and DHA drug group showed typical long rod-shaped structures with a smooth surface and an intact cell wall. In the DHA-CS NPs group and CS NPs group, the damaged cell morphology of the tested pathogens showed large surface collapse, abnormal cell breakage, and wrinkled cell walls. At present, some works clarified the destructive effect of CS on bacterial cell walls (Arkoun et al., 2017; Verlee et al., 2017) and there were no reports about DHA-CS NPs, so we chose GC-MS experiment to verify it.

### MTB Upregulated Fatty Acid Synthesis Pathway After DHA-CS NPs Treatment

**FIGURE 7:**
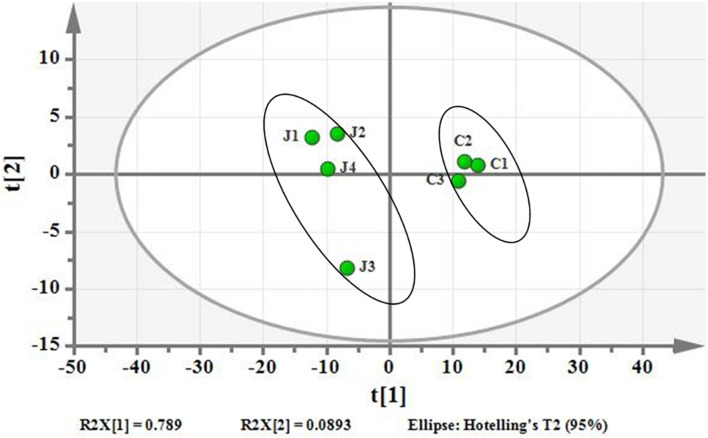
PCA differentiation using the GC-MS whole metabolome analyzed data of the individually cultured MTB in the absence (C) and presence (J) of 8.0 μg/ml DHA-CS NPs.

**FIGURE 8:**
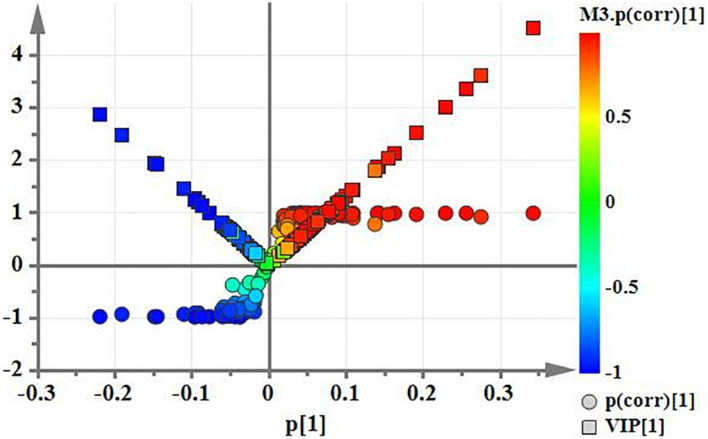
(V+S) plot of OPLS-DA model analysis selecting the differential metabolites of the individually cultured MTB in the absence and presence of 8.0 μg/ml DHA-CS NPs.

**FIGURE 9:**
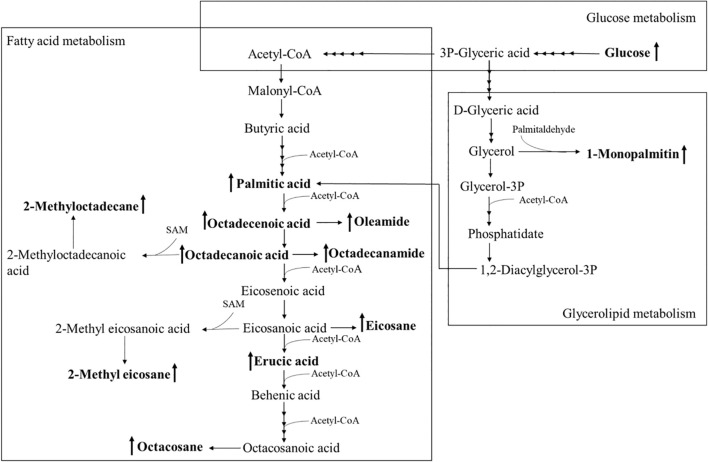
Altered MTB metabolome induced by treatment with 8.0 μg/ml DHA-CS NPs. The schematic representation indicates the 12 metabolite markers in bold, and increases in the metabolite markers are indicated by ↑.

Using PCA to analyze the two groups of samples, the results showed significant differences between individually cultured MTB samples in the presence and absence of DHA-CS NPs. The PCA score scatter plot in Figure 7 showed that the contribution rates of the two principal components were 78.0 and 9.0%, which could explain 95.0% of the data difference. Subsequently, based on the successful verification of the OPLS-DA and PLS-DA models, the metabolites with Variable Importance in Projection (VIP) value > 1.00, reliability correlation [p(corr)] value > 0.500 in the (V + S) scatter plot in Figure 8, and *t*-test *p*-value < 0.0500 were considered to be the differential metabolites with the most contribution (Table 2). Twelve differential metabolites obtained by the above multivariate statistical analysis were shown in Figure 9 in bold. These metabolites were related to MTB fatty acid synthesis pathway, glucose metabolism and glycerolipid metabolism.

**TABLE 2:** Metabolite markers that best explain the variance between the individually cultured samples in the absence and presence of DHA-CS NPs.

Name	^a^OPLS-DA (VIP)	^b^OPLS-DA [p (corr)]	^c^*t*-test (*p*-value)
Palmitic acid	4.50	0.984	0.0030
Octadecanoic acid	3.61	0.915	0.0060
Octadecenoic acid	3.00	0.991	0.0010
Oleamid	2.51	0.972	0.0010
1-monopalmitin	2.13	0.990	0.0010
Erucic acid	2.03	0.952	0.0010
Glucose	1.86	0.992	0.0010
2-methyl eicosane	1.43	0.988	0.0010
Eicosane	1.43	0.899	0.0060
Octadecane	1.42	0.952	0.0030
Octadecanamide	1.19	0.968	0.0020
2-methyl octadecane	1.05	0.927	0.0070

*^a^OPLS-DA (VIP): VIP-value > 1.00 are considered to be the differential metabolites with the most contribution.*

*^b^OPLS-DA [p (corr)]: correlation coefficient value > 0.500 are considered to be the differential metabolites with the most contribution.*

*^c^t-test (p-value): p-value < 0.0500 indicates that the difference was statistically significant.*

### DHA-CS NPs Combined With RFP Effectively Reduced MTB Resistance

The above results confirmed that DHA-CS NPs had a significant antibacterial effect on RFP-resistant strains. Next, we selected eighteen clinical isolates with known drug-resistant types to test whether DHA-CS NPs combined RFP can reduce the resistance.

**FIGURE 10:**
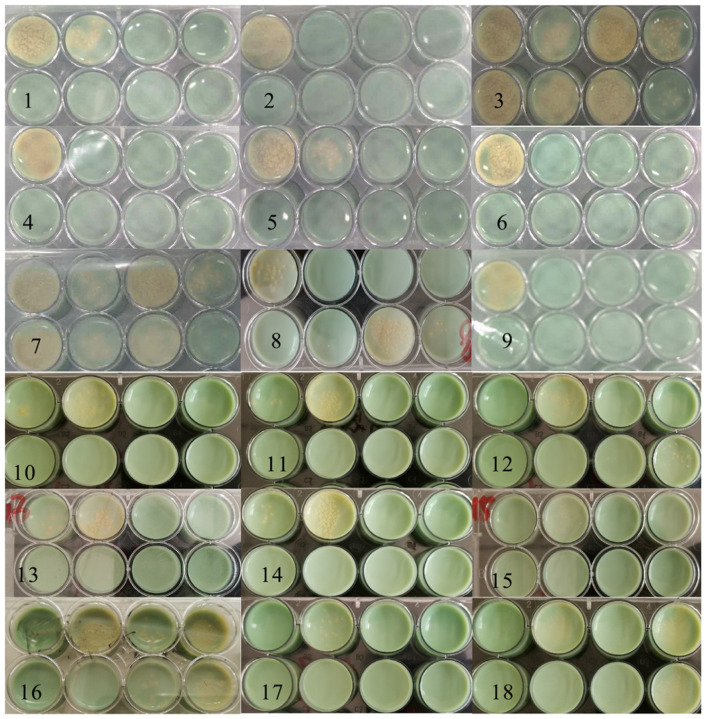
Solid medium drug susceptibility testing of 8.0 μg/ml CS-DHA NPs combined with anti-TB drugs (*n* = 18).

The testing was divided into two batches. In 1–9 plates, the concentration of the bacterial solution inoculated in “a” and “g” wells was 3.0 × 10^6^ CFU/ml, “b” and “h” with was 3.0 × 10^4^ CFU/ml, while the 10–18 plates were opposite. In Figure 10, the yellow colonies were MTB colonies. Colonies grew in the holes of the negative control groups. Still, no colonies grew in the high/low concentration inoculation holes of the drug groups, indicating that the drug resistance of the strain was reduced. Compared with the initial resistance to RFP, the resistance of 12 strains (1, 2, 4, 5, 6, 9, 10, 11, 13, 14, 15, and 17) was reduced by 8.0 μg/ml DHA-CS NPs combined with RFP, with an effective rate of 66.0%.

## Discussion

The DHA-CS NPs complex has the characteristics of DHA and CS. CS is particularly interesting as the broad spectrum of antibacterial activity of CS is well known and documented, offering the possibility of synergistic effects with antimicrobial drugs (Fu, 2018; Yin et al., 2019). DHA also has anti-MTB activity at a certain concentration (Kalani et al., 2019; Morake et al., 2019). In the bacterial live/dead testing, both CS and DHA have antibacterial effects on RFP-resistant MTB, and DHA-CS NPs have the best antibacterial action. Therefore, the significant antibacterial activity of DHA-CS NPs may be an additive effect of DHA and CS. However, its antibacterial effect is not positively related to the concentration. Appropriate low concentration of DHA-CS NPs has the most substantial antibacterial ability. The high concentration of CS probably can cause a certain amount of positive charge on the surface of the bacteria to keep the bacteria in suspension. In addition, due to the high concentration and high viscosity of CS, it has also coated the bacterial surface to prevent leakage of intracellular components. Therefore, the antibacterial effect of a high concentration of CS is achieved by isolating nutrients. Appropriate low concentration of CS may neutralize the negative charge on the surface of the MTB and make the bacteria glue together. In this way, DHA-CS NPs can easily penetrate the bacteria to disturb the cell membrane and cause cell death due to leakage of intracellular components (Sudarshan et al., 1992; Yang et al., 2007).

In a nutrient-rich environment, MTB is long rod-shaped and chain-shaped. After DHA-CS NPs treatment, acid-fast staining and auramine “O” staining has shown that the RFP-resistant MTB is short rod-shaped. It indicated that MTB growth is inhibited. The outer layer of the MTB cell wall is rich in lipid content, accounting for about 60.0% of dry weight. The thick lipid layer impedes penetration of Gram staining solution (Casabon et al., 2013; Vilchèze et al., 2014; Dong et al., 2016; Thompson et al., 2016; Zhou et al., 2016), which makes MTB challenging to stain. MTB of DHA-CS NPs group is easily stained, indicating that the DHA-CS NPs destroy the lipid structure of the outer layer of the MTB cell wall, making the Gram-dye solution enter it easier.

Metabolomics is an emerging “omics” science evolved from proteomics, genomics, and transcriptomics. It is based on dynamic changes in low molecular weight metabolites in organisms and has been applied in clinical research, human nutrition, medical research, and development, microbiology metabolism and mechanism researches. This experiment used metabolomics to confirm that RFP-resistant MTB fatty acid synthesis and cell wall repair increased after DHA-CS NPs treatment. Twelve differential metabolisms are identified by GC-MS technology, and 10 of these metabolites are directly related to MTB fatty acid synthesis, including palmitic acid, octadecenoic acid, octadecanoic acid, erucic acid, and so on. These metabolites can form methyl-branched chain fatty acids and eventually form mycolic acid, which is a crucial component of MTB cell wall (Preez and Loots, 2012; Koen et al., 2018). The elevation of these metabolites suggests that MTB upregulates the cell wall synthesis pathway, based on the increased concentration of alkanes (eicosane, octacosane, 2-methyl octadecane, 2-methyl eicosane) and fatty acid amides (oleamide and octadecanamide). Considering that MTB treated with DHA-CS NPs requires more energy to preferably utilize fatty acids toward cell wall repair, MTB will use glucose as a primary energy substrate to obtain energy (Carvalho et al., 2010). In this work, the increase in glucose concentration confirmed it. In addition, the intermediate product of glycolysis can also be used as a substrate for MTB fatty acid biosynthesis, such as 3P-glyceric acid, which can be confirmed by the increase in the concentration of 1-monopalmitin.

At present, although there is no report on the anti-MTB mechanism of DHA-CS NPs, studies have reported that artemisinin drugs and CS can destroy the cell wall structure of bacteria or parasites. Artemisinin and its derivatives can generate free radicals under the mediation of Fe^2+^ and destroy the cell membrane of Plasmodium (Clark et al., 1984; Meshnick, 2002; Antoine et al., 2014). Therefore, the rupture of DHA-CS NPs peroxy bridge may also generate free radicals to attack the bacterial cell wall. CS is an N-deacetylation product of chitin and is the positively charged polyelectrolyte in polysaccharides (Guo, 2007; Tantala et al., 2019). Studies have shown that it can combine with negatively charged cell walls, thereby exerting a broad-spectrum antibacterial effect (Fu, 2018; Yin et al., 2019). Because the cell wall of MTB is negatively charged, DHA-CS NPs can bind to it and interfere with cell wall synthesis. In summary, the antibacterial mechanism of DHA-CS NPs on RFP-resistant MTB may be that it interferes with cell wall synthesis and generates free radicals to damage the cell wall.

The results of DHA-CS NPs combined with anti-tuberculosis drugs have shown that 8.0 μg/ml DHA-CS NPs can reduce MTB resistance. Currently, there are some reports of DHA reduce bacterial resistance. Wu et al. (2013) have shown that the combination of DHA and ampicillin can reduce the resistance of *E. coli.* Lv et al. (2009) have found that artemisinin combined with cefoxitin can reduce the resistance of *Staphylococcus aureus.* Recent studies have demonstrated that efflux pumps of MTB provide a crucial mechanism in the drug-resistant development of anti-TB drugs (Parumasivam et al., 2016). The above results confirm that DHA-CS NPs can destroy the MTB cell wall, so the accumulation of RFP in the cell may be one reason for the decreased resistance. RNA polymerase (RNA-PA) is an essential enzyme required for bacterial transcription. RFP can inhibit gene transcription by binding to the beta subunit of the DNA-dependent RNA-PA, encoded by the *rpoB* gene. When *ropB* gene is mutated, the binding site of RFP and RNA-PA disappears, leading to MTB resistance. In previous experiments, gene chip results have shown that DHA-CS NPs decrease the *ropB* mutant gene level (Zhang, 2013). It also may be the reason for the reduced resistance to RFP.

Overall, DHA-CS NPs reduced the resistance of MTB to RFP, possibly because it reduced the *ropB* mutant gene level or damaged MTB cell wall to increased RFP intake. Therefore, the combination of DHA-CS NPs and the classic anti-tuberculosis drugs RFP is expected to constitute an effective treatment for TB.

## Conclusion

In conclusion, DHA-CS NPs showed enhanced antibacterial activity compared with free DHA and CS, which was based on the additive effect of DHA-CS NPs by effectively destroying the cell wall of MTB. Furthermore, at effective concentrations, DHA-CS NPs can reduce the RFP resistance, making it a potential anti-TB drug worthy of further investigation.

## Data Availability Statement

The original contributions presented in the study are included in the article/Supplementary Material, further inquiries can be directed to the corresponding author/s.

## Author Contributions

XG: conceptualization, data curation, software, and writing–original draft preparation. QC: methodology. YanZ and XG: validation. YalZ: formal analysis, investigation, resources, and project administration. PH: writing–review and editing. ZJ: visualization and supervision. All authors have read and agreed to the published version of the manuscript.

## Conflict of Interest

The authors declare that the research was conducted in the absence of any commercial or financial relationships that could be construed as a potential conflict of interest.

## Publisher’s Note

All claims expressed in this article are solely those of the authors and do not necessarily represent those of their affiliated organizations, or those of the publisher, the editors and the reviewers. Any product that may be evaluated in this article, or claim that may be made by its manufacturer, is not guaranteed or endorsed by the publisher.

## Supplementary Material

Supplementary Figure 1 MTB solid drug-sensitive culture plate.
