# Supplementary figures and images for: Dihydroartemisinin-Loaded Chitosan Nanoparticles Inhibit the Rifampicin-Resistant Mycobacterium tuberculosis by Disrupting the Cell Wall
Source: Front Microbiol. 2021 Sep 22;12:735166. doi: 10.3389/fmicb.2021.735166 (PMC8500176; doi:10.3389/fmicb.2021.735166)

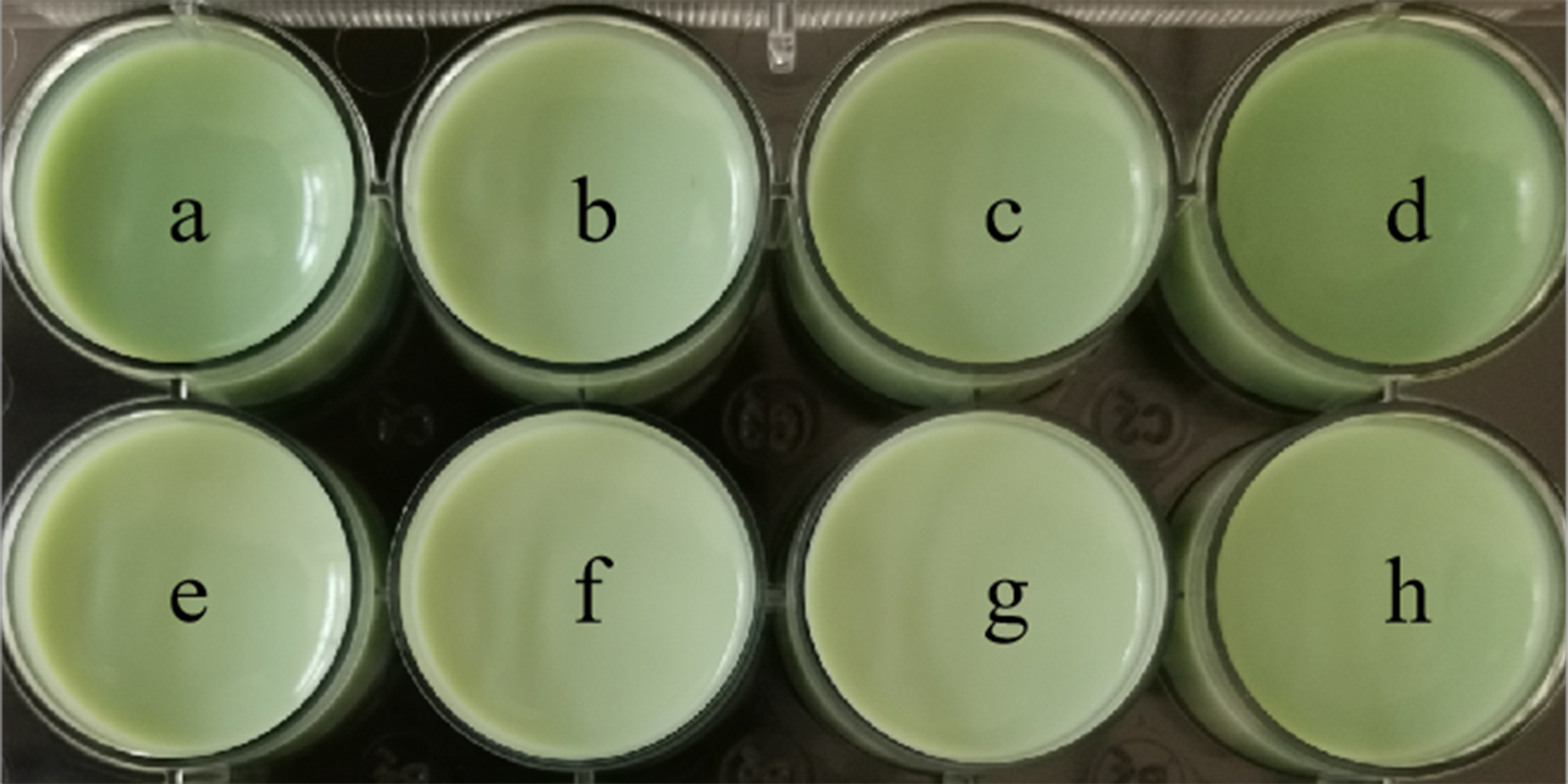

Supplement: Supplementary Figure 1 — MTB solid drug-sensitive culture plate. [file Image_1.tif]
